# Observation system for the technical-tactical analysis of judo by the Rio 2016 Olympic champions

**DOI:** 10.1371/journal.pone.0303689

**Published:** 2024-05-20

**Authors:** David Soriano, Rafael Tarragó, Daniel Lapresa, Mike Callan, Xavier Iglesias

**Affiliations:** 1 Institut Nacional d’Educació Física de Catalunya (INEFC), Universitat de Barcelona (UB), Barcelona, Spain; 2 Universidad de La Rioja, Logroño, Spain; 3 University of Hertfordshire, Hatfield, United Kingdom; Pontifical University of Salamanca: Universidad Pontificia de Salamanca, SPAIN

## Abstract

An observational methodology system has been designed which allows the observation and analysis of the technical-tactical behaviour and interaction of *judokas* during competition. The observation instrument (JUTACTIC) is composed of 8 fixed criteria that provide information related to the competition and the competitors and 13 variable criteria that, throughout the intrasessional monitoring of each combat, allow the behaviour displayed by both judokas and their interaction to be recorded. From an observational sample consisting of matches from the Rio 2016 Olympic champions and the corresponding samples made using the LINCE PLUS software, evidence of validity, reliability, generalizability and applicability of the observation system is provided. The content validity of the observation instrument has been endorsed by a panel of experts (n = 11). Intra and inter-observer reliability has been guaranteed from the results obtained in the Fleiss Kappa and the Krippendorff Alpha. The generalizability analysis with the design structure [Category] [Participants] / [Matches] has confirmed that around seven matches are needed to accurately analyse the behaviour of the competitor under study. The practical application possibilities of the observation instrument has been shown with an example of the results obtained and the regular behaviour structures detected (T-patterns) using the THEME software.

## Introduction

Judo is an Olympic combat sport of Japanese origin which includes gripping, with direct opposition from the opponent. For men judo first appeared in the 1964 Tokyo Olympic Games, it has featured in every Summer Olympic Games since 1972. For women, judo was a demonstration sport in 1988 Seoul, and a full sport from 1992 in Barcelona.

Techniques are applied which can be throwing from a standing or sacrifice position, or holds, armlocks or strangles with both *judoka* (judo practitioners) fighting on the ground [1]. At the Rio Olympic Games, the contest area of the tatami was 10 metres x 10 metres. There was a 4 metres safety area.

This sport is structured into effort sequences. In Rio 2016 the matches lasted five minutes for men and four for women, with a golden score in case of a tie. This extra phase does not end until one of both judokas scores. The fight begins standing up, and after the referee’s hajime (start call) both judoka begin a dynamic and active phase, in which they move around the tatami (mat area) looking for the opportunity to grab their opponent [2], this phase constitutes the time to attempt the grip (kumi-kata). Next, there is the phase of establishing the grip, which is static and essential for the next phase; that of applying a throwing technique (nage-waza) [3]. Sometimes these strenuous sequences continue into ground fighting (ne-waza). For various reasons stipulated by the rules of the International Judo Federation (2015), it is possible that the referee decides to pause the match by a call of matte, without a score having been made. This produces a pause, the moment in which the athletes go to the centre of the tatami to resume the match. This brief interlude can be used by coaches to give technical-tactical instructions and by judoka to analyse or reflect on other considerations of their own. Hence, this period of time, the matte–hajime phase, becomes of vital strategic importance [4].

Analysing the existing literature, it is evident that judo has been one of the most studied combat sports in recent years, dealing with topics as diverse as: the relationship between technique, tactics and performance [5], time-motion and competitive judo analyses [4, 6–9], judo learning [10], coaching [11], *kumi-kata* analysis [1, 12, 13], biomechanics [14], anthropometry [15], and others.

Related to this research, there are previous studies on the type of grip, laterality and effectiveness [13], and temporal structure of judo combat [10]. Also, studies related to the detection of T-Patterns in the performance of different technical actions of judo [10] and the search for T-Patterns related to the temporal structure in judo with visual impairment [16].

Considering that judo has a large number of technical elements to consider in a wide variety of specific situations [1, 9], to aspire to success in high-level judo, it is necessary for coaches and athletes to carry out combat analysis both of their own technical-tactical performance and that of their rivals [9, 17]. As has been mentioned, there are other observational instruments for the analysis of judo combats [9], but none of them allow the detection of regular behaviour structures in the interaction between *judoka*. This allows the generation of reports on athletes or rivals in a specific way, dependant on the requirements in the competition.

The purpose of this study is to design an observational tactical analysis instrument that provides evidence of validity, reliability, generalization and applicability, allowing the characterization of technical-tactical behaviour during combat in Judo. A second objective was to verify its practical usefulness. The Olympic judo champions at the 2016 Rio Olympics were analysed [18].

## Method

This study has been conducted using observational methodology, since it satisfies the fundamental requirements of: spontaneity of behaviour; which takes place in natural contexts; temporal continuity, which enables intra-sessional monitoring and the subsequent performance of diachronic behaviour analyses; behavioural perceptiveness; and in the absence of standard instruments which allow satisfaction of the predetermined objective, the design of an observational instrument built *ad hoc*

According to Anguera et al. [19], the observational design is: nomothetic -the two competitors are analysed differentially-; intersessional monitoring -different matches are analysed- and intrasessional -the different actions carried out during the match are recorded- which allows diachronic analyses of behaviour to be carried out; and multidimensional -as reflected in the different criteria that constitute the observation instrument.

### Participants

The observational sampling carried out in this study consists of the matches of six judoka who became Olympic champions in Rio 2016 (in the female category in the weights of -48kg, -63kg and +78kg and in the male category in the weights of -60kg, -81kg and +100kg), from August 6 to 12. The categories chosen are the lowest, intermediate, and highest weights. In total there were 42 matches observed. For each judoka this was five matches taken from the IJF World Tour Olympic Qualification period and the Olympic Games semi-final and final matches. In total, seven fights for each athlete have been analysed. The recruitment period was from 17 April 2018 to 7 February 2019.

The videos of the matches corresponding to the qualifying tournaments have been obtained from the portal of the International Judo Federation (IJF) where they are freely available, as well as public television broadcasts of the Rio 2016 Olympic Games. The matches were recorded from start to finish, without interruptions or image editing.

The ethical approval was obtained from the Clinical Research Ethics Committee of the Sports Administration of Catalonia, Spain (registry number: 00998/2912/2010), and the protocol were written according to the standards established by the Declaration of Helsinki. Informed consent of the participants is not required when it comes to public broadcasting images in accordance with the Ethical Principles of the American Psychological Association [20].

### Observational instrument

An *ad hoc* observational instrument -composed of 21 criteria, 8 fixed and 13 variable- (JUTACTIC) has been designed whose general structure is that of a field format and in which category systems have been nested in each of the criteria, which meet the conditions of exhaustiveness and mutual exclusivity [19]. The content validity was guaranteed through the consistency and conceptual robustness of the judo theoretical framework, as well as through the critical evaluation of the observational instrument by a panel of experts (N = 11). The experts were national judo trainers with a minimum third grade *dan*, calculating the percentage of positive matches in their evaluation of the criteria of JUTACTIC using the “R” software (Studio Version 1.4.1717, PBC). Tables 1 and 2 presents the summary version of the observation instrument. Table 1 shows the fixed criteria in the registration of each combat. Table 2 shows the variable criteria in the intrasessional monitoring of each match. The observation instrument is made up of 8 fixed criteria that provide information related to the competition and the competitors and 13 variable criteria that, throughout the intrasessional monitoring of each combat, allow the behaviour displayed by both judokas and their interaction to be recorded. We define the kumi kata at the right moment of action 1 of each judoka per unit of observation.

**Table 1:** Fixed criteria, categories and basic codes of the observation instrument.

Criteria	Categories and codes
SEX	M (male) F (female)
LEVEL	INT (international), STA (state), REG (regional)
PHASE	FIN (final), SEMI (semi-final), 3&5, 1/4, 1/8, REP (repechage)
WEIGHT CATEGORY MALE	M60, M66, M73, M81, M90, M100, M+100
WEIGHT CATEGORY FEMALE	F48, F52, F57, F63, F70, F78, F+78
CHAMPION’S JUDOGI COLOR	WC (white champion), BC (blue champion)
RIVAL’S JUDOGI COLOR	WR (white rival), BR (blue rival)
LATERALITY KUMI KATA	RR (both right handed), RCLO (righthanded champion and left-handed rival), LCRO (lefthanded champion and right-handed opponent), LL (both left handed)

*Note*. This table shows the summary version of the observation instrument (JUTACTIC) of our study.

**Table 2:** Variable criteria, categories and action codes of the observation instrument.

SCORE	IC (*ippon* champion), WC (*waza-ari* champion), YC (*yuko* champion), NC (nothing champion), IR (*ippon* rival), WR (*waza-ari* rival), YR (*yuko* rival), NR (nothing rival).
KUMI KATA PREVIOUS ACTION 1, 2, 3, 4	KKC 1 HAND R S (one hand, right handed in sleeve), KKC 1 HAND R L (one handed, right handed in lapel), KKC 1 HAND L S (one handed, left sleeve), KKC 1 HAND L L (one handed, left lapel), KKC 2 SLEEVES (two sleeves), KKC 2 HANDS HIGH (2 hands, sleeve and high grip), etc.
ATTACK DIRECTION_ACTION 1, 2, 3, 4	BR_1 (back right), BL_1 (back left), FR_1 (front right), FL_1 (front left), BRDIAG_1 (back right diagonal), BLDIAG_1 (back left diagonal), RFDIAG_1 (right front diagonal), FLDIAG_1 (diagonal front left), RL_1 (right lateral), LL_1 (left lateral)
POSITION NE-WAZA_ACTION 1, 2	CFUGRL_1 (face-up champion grabs rival’s leg with his legs), CQ_RA_1 (quadruped or stretched champion, rival attacks), CBL_RFU_1 (champion between legs of rival who is face up), etc.
ACTION 1 (I), 2 (II), 3 (III), 4 (IV)–TE-WAZA	SON_I_C, SON_I_R (*seoi-nage*, C of Champion or R of Rival), ISN_I_C, ISN_I_R (*ippon-seoi-nage*), SOO_I_C, SOO_I_R (*seoi-otoshi*), TOS_I_C, TOS_I_R (*tai-otoshi*), etc.
ACTION 1 (I), 2 (II), 3 (III), 4 (IV)–KOSHI-WAZA	UGO_I_C, UGO_I_R (*uki-goshi*), OGO_I_C, OGO_I_R (*o-goshi*), KOG_I_C, KOG_I_R (*koshi-guruma*), TKG_I_C, TKG_I_R (*tsurikomi-goshi*), STG_I_C, STG_I_R (*sode-tsurikomi-goshi*), etc.
ACTION 1 (I), 2 (II), 3 (III), 4 (IV)–ASHI-WAZA	DAH_I_C, DAH_I_R (*de-ashi-harai*), HIZ_I_C, HIZ_I_R (*hiza-guruma*), STA_I_C, STA_I_R (sasae-tsurikomi-ashi), OSG_I_C, OSG_I_R (o-soto-gari), OUG_I_C, OUG_I_R (o-uchi-gari), etc.
ACTION 1 (I), 2 (II), 3 (III), 4 (IV)–MA-SUTEMI-WAZA	TNG_I_C, TNG_I_R (*tomoe-nage*), SUG_I_C, SUG_I_R (*sumi-gaeshi*), HKG_I_C, HKG_I_R (*hikikomi-gaeshi)*, etc.
ACTION 1 (I), 2 (II), 3 (III), 4 (IV)–YOKO SUTEMI-WAZA	YOT_I_C, YOT_I_R (*yoko-otoshi*), TNO_I_C, TNO_I_R (*tani-otoshi*), HNM_I_C, HNM_I_R (*hane-makikomi*), etc.
ACTION 1 (I), 2 (II), 3 (III), 4 (IV)–OSAEKOMI-WAZA	KEG_I_C, KEG_I_R (*kesa-gatame*), KKE_I_C, KKE_I_R (*kuzure-kesa-gatame*), UKG_I_C, UKG_I_R (ushiro-kesa-gatame), etc.
ACTION 1 (I), 2 (II), 3 (III), 4 (IV)–SHIME-WAZA	NJJ_I_C, NJJ_I_R (*nami-juji-jime*), GJJ_I_C, GJJ_I_R (*gyaku-juji-jime*), KJJ_I_C, KJJ_I_R (*kata-juji-jime*), etc.
ACTION 1 (I), 2 (II), 3 (III), 4 (IV)–KANSETSU-WAZA	UGR_I_C, UGR_I_R (*ude-garami*), JGT_I_C, JGT_I_R (ude-hishigi-juji-gatame), UGA_I_C, UGA_I_R (*ude-hishigi-ude-gatame*), HIG_I_C, HIG_I_R (*ude-hishigi-hiza-gatame*), etc.
WINNER	WC, WR (winning champion or rival)

*Note*. This table shows the summary version of the observation instrument (JUTACTIC) of our study.

JUTACTIC was designed following the Rules of Organization and Sport (SOR) document [18] of the International Judo Federation (2015). The penalty rules corresponding to the 2015 IJF regulations apply, the year in which the registered competition was held (Rio Olympic Games). The corresponding score or Shido was marked on the record sheet as decided by the referee. In the event of a tie the golden score was carried out.

### Data recording and coding

The registration and coding of the data has been carried out using the LINCE PLUS 1.3.2 software [21]. The recorded data has been exported directly from the LINCE PLUS to the T-pattern detection program, THEME 6EDU [22]. According to Bakeman’s [23] classification, the data obtained is time-based and concurrent (type IV).

We have considered as an observation unit the different combat actions and situations that provoked arbitration decisions (*hajime*, *matte*, sanctions, score…), as well as the situations that, from *tachi-waza*, generated *nage-waza* actions or passed to *ne-waza* without scoring changes or arbitration decision.

In each observation unit, the multidimensional analysis was carried out in which the categories indicated in the observation instrument were recorded (Tables 1 and 2).

The multidimensional record of each observation unit generated each of the lines of our analysis matrix.

### Data quality control

The quality of the data was determined based on the evidence provided of reliability and generalizability, using the process below.

#### a) Agreement between observations

There were three expert observers in charge of the registry. Each observer had over 20 years of experience in the judo as practitioners, minimum third *dan* and more than 10 years as coaches. All the observers underwent a training process in the understanding and use of the observational instrument. The primary observer recorded all the matches. Both the primary observer and the other two observers recorded three bouts on two occasions for reliability analysis.

#### b) Generalizability of the results

This section was performed within Generalizability (TG) [24] and from the Works of Blanco-Villaseñor [25]. The theory of Generalizability allows us to evaluate the different sources of variability (or facets) of a design, allowing us to determine what size each of them must have to obtain a high precision score—a generalizability coefficient close to 1 -. The sum of squares necessary for the Generalizability design has been obtained using SPSS, version 24. Subsequently, the data has been entered into the Software Generalizability Theory (GT). Due to its relevance (how many matches do we need to discover the behaviour of a competitor?) the design [Category] [Participants] / [Matches] have been carried out, within the General Linear Model (GLM), from which the option offered by SPSS of data types III (for data which has not been taken randomly). The estimation of the variance components has been performed in an infinite random way for the three facets (categories with 197 levels, participants with 6 levels, and combats with 7 levels). In this design it has not been necessary to carry out the optimization plan, as very high values of generalization precision were obtained with the seven analysed bouts of each judoka.

### Data analysis

Contingency tables were made and the chi-square was analysed to determine the existence of a significant association between the records in the different categories of analysis according to their efficacy values, differences between participants or by groups of men and women.

To detect regular behaviour structures in the technical-tactical behaviour of athletes in competition (own or rival) and their interaction, one of the most widely used diachronic analysis techniques has been used for its informative potential in observational methodology: detection of T-patterns using the THEME software (v.6 Edu) [22, 26]. Although the main contribution of THEME is the detection of temporal patterns, the software also offers the possibility of detecting sequential structures under the order parameter from an assignment of a constant duration to each behaviour unit. This provides some very relevant possibilities for the analysis of sequentiality, since it allows us to deduce if the behaviours are consecutive or if there are gaps in the T-pattern ‘interspersed behaviours’ between the detected multi-events [27].

The following search parameters have been selected: a) the type T-patterns free has been used; b) a minimum number of occurrences equal to or greater than 2 has been set; c) the level of significance has been established at p<0.005, that is, the percentage of accepting a critical interval due to chance is 5%.

Once the search was carried out by applying these quantitative filters for the selection of T-patterns, different search options were applied as qualitative filters based on the activation of relevant dimensions for the T-patterns. Combat analysis: a) regular structures of behaviour that incorporate information regarding the directions of the attack actions used b) offensive technical actions and their effectiveness.

## Results

The analysis of the results reflects that 607 combat actions have been analysed with average values of 14.5 ± 6.2 actions per combat in a range of 1 to 25. For our study, we determined that a combat action was each of the techniques performed by the *judoka*, whether or not they had a score from the referee. Within the same attack sequence, the different possibilities of combinations and/or counterattacks were considered. They are reflected in our study as Action-1 being the first attack and Action-2, etc. as the successive ones, and it is also recorded if they are from one judoka or another. It is understood that if these actions occur it is because there is an interaction within the same observation unit. With which it is essential that the viewer of the videos is a technician specialized in judo, with experience and training in the *ad hoc* instrument designed and in the LINCE PLUS program. Of the total actions, in 461 there is a statement by the referee about the score or not of the action, and the remaining 146 are transition actions without arbitration.

We have focused the descriptive analysis on these 461 actions, of which 88.7% are carried out as *nage-waza* actions (throwing techniques) and 11.3% are *katame-waza* actions (grappling techniques), with the proportion of work on the r ground being higher in women (16%) than in men (6%) (p<0 .001), without differences (n.s.) in the different weight categories in each sex. Only 9.3% of the actions (N = 461) in which the referee intervenes are effective on the scoreboard, in 7.6% of the times favourable to the champion and the remaining 1.7% to the opponent, with no significant differences between male and female competitors.

Of the 461 combat actions analysed, in 396 the first action is performed in *tachi-waza* (standing techniques), while only 12 start in *ne-waza* (ground works), leaving the rest in preparation positions. The male *judoka* starts their judo actions standing differently from the women (p<0.001), using the 55.2%*ashi-waza* (39.6% women), 26.3% *te-waza* (33.2% women), 11.3% *sutemi-waza* (7.9% women) and 7.2% *koshi-waza* (19.3% women). In relation to the weight categories, men do not present significant differences, but women do. If we look at the effectiveness on the scoreboard based on which foot judo actions have started the attack phase, we see how *sutemi-waza* is the most effective with 15.8% of scores, followed by *te-waza* (10.2%), *ashi-waza* (8.6%) and, finally, *koshi-waza* (5.7%), with no differences based on gender or combat category. This is an important aspect to consider and interesting to study more deeply. Since it gives us relevant information at a technical-tactical level. The fact that the most used techniques in women’s judo are *sutemi-waza*, offers us the opportunity to better prepare tournaments in that category.

We observed how the *kumi-kata* is used differently depending on the gender of the champions (p<0.001), being for example the one with sleeves and high above the shoulder (KUMI KATA CHAMPION 2 HANDS HIGH, 11.9%) more used in men than in women (5.0%). For their part, women use more of the classic sleeve and lapel grip (KKC 2 HANDS C, women 14.5%; men 5.4%). Differences (p<0.001) are also observed in the prioritization of the use of the different kumi kata according to the athletes.

Of the champion’s kumi kata analysed, the most effective for the champion’s score (p<0.01) were the high cross grip (KKC CROSS HIGH, 33.3% of N = 6), the right hand grip on the sleeve (KKC 1 HAND RIGHT SLEEVE, 15.0% of N = 20), right hand grip on flap (KKC 1 HAND RICHT FLAP, 12.8% of n = 39) and high two-hand grip (KKC 2 HANDS HIGH, 12.8% of N = 78).

The champion is also more effective (p<0.05) when his opponent uses a left hand *kumi-kata* on the sleeve (KKR 1 HAND LEFT SLEEVE, 18.2% of N = 22), when he uses a left-hand grab on the lapel (KKR 1 HAND LAPEL LEFT, 17.5% of N = 40) and when using a waist and sleeve *kumi-kata* (KKR WAIST AND SLEEVE, 33.3% of N = 3).

In the following sections, the results are presented which will serve as evidence of the validity, reliability, generalizability and applicability of the observational system designed to characterize the technical-tactical behaviour of *judoka* in combat.

### Content validity

The panel of experts (N = 11) assessed the observational instrument JUTACTIC showing their agreement or disagreement for each criterion and category. The results showed 8.258 positive judo matches out of 10.206 possible, which is a percentage of 80.9% positive matches. To obtain the 95% confidence intervals, we apply the binom.test function of R, resulting in between 0.801 and 0.817.

### Reliability. Agreement between observations

The calculation of intra-observer and inter-observer reliability (Table 3) was performed from Fleiss’s Kappa [28] and Krippendorff’s Alpha, using the "R" software (RStudio Version 1.4.1717, PBC). Criteria with no occurrences in the reliability analysis are discarded.

**Table 3:** Results of inter and intra-observation reliability.

	Intra-observation		Inter-observation	
	Kappa	Alpha (Krippendorff)	Kappa	Alpha (Krippendorff)
	(Fleiss)		(Fleiss)	
LATERALITY KUMI KATA	1	1	1	1
CHAMPION MARKER	1	1	1	1
RIVAL MARKER	NA	1	NA	1
DURATION	0.9564	0.9567	0.9855	0.9855
CONTEST TIME	0.9110	0.9116	0.8233	0.8237
STANDING OR GROUND	1	1	0.8795	0.8798
SITUATION ON THE MAT	1	1	0.9130	0.9132
SCORE	0.9726	0.9728	0.9441	0.9444
SHIDO CHAMPION	0.9191	0.9196	0.8419	0.8422
SHIDO RIVAL	0.9191	0.9196	0.8419	0.8422
KUMI KATA CHAMPION PREVIOUS ACTION 1	1	1	0.9268	0.9270
KUMI KATA PREVIOUS RIVAL ACTION 1	0.9524	0.9527	0.9509	0.9510
ATTACK DIRECTION_ACTION 1	0.9762	0.9764	0.9008	0.9010
POSITION NE WAZA_ACTION 1	1	1	0.8244	0.8248
ACTION 1—TE WAZA	1	1	1	1
ACTION 1—KOSHI WAZA	1	1	1	1
ACTION 1—ASHI WAZA	1	1	0.9202	0.9204
ACTION 1—SUTEMI WAZA	1	1	1	1
ACTION 1—OSAEKOMI WAZA	1	1	1	1
ACTION 1—SHIME WAZA	1	1	0.6633	0.6640
ATTACK DIRECTION_ACTION 2	1	1	0.8891	0.8893
POSITION NE WAZA_ACTION 2	1	1	1	1
ACTION 2—KOSHI WAZA	1	1	1	1
ACTION 2—ASHI WAZA	1	1	1	1
ACTION 2—SUTEMI WAZA	1	1	1	1
ACTION 2—OSAEKOMI WAZA	1	1	1	1
WINNER	1	1	1	1

*Note*. This table presents the results of the analyzes of inter and intra-observation reliability.

### Generalizability analysis

The high value of the coefficient of determination r^2^ obtained (r^2^ = 1) indicates that with the combination of the chosen facets we can explain with guarantees the variability that the data packages which constitute the observational sampling contribute to its development. The analysis of the generalizability coefficients in the design structure [Category] [Participants] / [Matches] determines that a generalization accuracy reliability of 0.928 is achieved -same value of the relative generalizability coefficient (e2) and the absolute (Φ)-. This result allows us to verify the homogeneity of the fights carried out by each of thesubjects(competitors) and informs us that as coaches we need around 7 fights to ‘capture’ with guarantees the competitor behaviour which is interesting to analyze in competition.

### Records obtained through the observation instrument

Each of the records obtained by means of the observational instrument under development suppose the overturning of the reality of the behaviour displayed in a judo combat by a competitor, his rival or the interaction between both. As an example of the practical application possibilities of the observational instrument and its practical possibilities for judo coaches, the record corresponding to the matches fought between different competitors is presented. As can be seen in Table 4, the succession of rows in the record is carried out diachronically, under the order parameter. As an example of the applicability of the observational instrument and its practical possibilities for judo coaches, the record corresponding to the final of theunder 81 kg category of the Rio 2016 Olympic Games between Stevens (USA) and Khalmurzaev (Russia) is presented. As can be seen in Table 4, the succession of rows in the record is carried out diachronically, under the order parameter.

**Table 4:** Example of a record obtained by means of the observation instrument JUTACTIC, corresponding to one particular combat.

Order	Multievent
1	HAJ,WINGC,TI,TW
2	WINGC,TI,TW,CCENTER,NR,KKC 2 MANOS C,KKR 2 HANDS C,FR_1, TOS_I_R
3	MAT,WINGC,TI,TW,RCENTER,NR,KKC 1 MANO I S, KKR 1 HAND R L, FR_1, STA_I_R
4	HAJ,WINGC,TI,TW
5	MAT,WINGC,TI,TW,RCENTER,NR,KKC 2 HANDS C,KKR 2 HANDS C,RL_1,DAB_I_R,LI_2,KUG_II_R
6	WINGC,TI,TW,SR PS19
7	HAJ,WINGC,TI,TW
8	MAT,WINGC,TII,TW,RCENTER,NC,KK 2 HANDS C,KKR 2 HANDS C,RL,1,DAB_I_C,DI_2,UMA_II_C
9	HAJ,WINGC,TII,TW
10	MAT,WINGC,TII,TW,RCENTER,IC,KKC 2 HANDS C,KKR 2 HANDS C, DI_1,UMA_I_C

*Note*. This table shows us the results obtained from the observation of a competitive judo match.

We can see how in row 1 the referee starts the fight with *hajime* (HAJ), won by the champion (WC), and that in minute 1 (TI) the actions are performed in standing judo (TW, *tachi-waza*). Row 2 shows how in minute 1 (TI) in *tachi-waza* (TW) the attack action is performed by the opponent from the center of the *tatami* (RCENTER) without scoring (NR), with a grab prior to the attack classic two hands of both the champion (KKC 2 HANDS C) and the rival (KKR 2 HANDS C) through *tai-otoshi* (TOT_I_R) directed to the right forward (FR). And so the rest of the rows can be successively trancribed, until in row 10 the referee ends a match (MAT) won by the champion (WC) in minute 2 (TII); performing a first action in the center of the *tatami* (CCENTER); obtaining *ippon* (IC) with a classic two-handed sleeve and flap grab by the champion (KKC 2 HANDS C) and the rival (KKR 2 HANDS C), by means of *uchi-mata* (UMA_I_C) directed forward and left (FL).

### T-patterns detected

The observation instrument allows capturing regular behaviour structures in the recordings corresponding to one or several competitors. It is important to keep in mind the structure of a sequence in standing judo from previous works [4]: *hajime*—move without contact—fight for *kumi-kata*—established *kumi-kata*—attack—score—*matte*.

The following is an example of the possibilities of the observation instrument for the analysis of different dimensions of combat, the T-patterns detected from different search options.

Thus, in the first place, the aim is to capture the conduct performed by a competitor in the form of behaviour patterns (Table 5) which incorporate in their constitutive clusters the directions in which they carry out the offensive technical actions. According to the information contained in the observation instrument (Tables 1 and 2), this search option is formulated: direction of attack 1—action or technique 1—direction of attack 2—action or technique 2—direction of attack 3—action or technique 3—direction of attack 4—action or technique 4.

**Table 5:** T-patterns detected in the competitor (Khalmurzaev) which incorporate in their constitutive clusters the directions in which they carry out the offensive technical actions.

ID	Pattern	Occurrences	Combats	Average intern intervals
1	((FL_1,ISN_I_R FL_1,ISN_I_R) (BL_1,OSG_I_C FL_1,ISN_I_R))	2	1–6	6.5-5-3
2	((FL_1,ISN_I_R FL_1,ISN_I_R) (FL_1,ISN_I_R FL_1,ISN_I_R))	2	1–6	6.5-8-11.5
3	(BL_1,OSG_I_R (FL_1,ISN_I_R FL_1,ISN_I_R))	2	6–6	3–7
4	(BR_1,UMA_I_R (FL_1,ISN_I_R FL_1,ISN_I_R))	2	1–1	2.5–11
5	((FR_1,STA_I_C BL_1,OSG_I_C) LL_1,SON_I_R)	2	1–2	5–17
6	(BL_1,DAB_I_R FR_1,STA_I_C)	2	5–5	4
7	(BL_1,KGU_I_C BL_1,OSG_I_C)	2	1–2	8.5
8	(BL_1,OSG_I_C FL_1,ISN_I_R)	3	1-1-6	2.33
9	(BL_1,OSG_I_R FL_1,ISN_I_R)	2	6–6	3
10	(FR_1,TOT_I_R FL_1,UMA_I_R)	2	3–4	5
11	(FR_1,UMA_I_R FL_1,ISN_I_R)	2	1–1	2.5
12	(FL_1,ISN_I_R FL_1,ISN_I_R)	6	1-1-1-6-6-6	8.67
13	(FL_1,UMA_I_C FR_1,ISN_I_C)	2	2–3	10.5

*Note*. This table shows us the results obtained at the level of behaviour patterns related to the directions of attack and the techniques used. The identifier that differentiates each of the T-patterns detected is presented, its pattern in chain format—constitutive multi-events -, the number of occurrences that the T-pattern has and in which combats they take place, as well as the average of the internal intervals between the constituent multievents.

Secondly, for the T-patterns detection procedure, the dimensions which allow us to characterize the effectiveness of the technical actions carried out by the competitor, incorporating the attacks carried out and their score, have been activated. This search option is formulated: score—action or technique 1—action or technique 2—action or technique 3—action or technique 4. Table 6 shows an example of the T-patterns detected.

**Table 6:** T-patterns detected in the competitor (Khalmurzaev) which incorporate the attacks carried out and their score into their constitutive clusters.

ID	Pattern	Occurrences	Combats	Average intern intervals
1	((NC,STA_I_C NC,OSG_I_C) (NC,STG_I_C NR,SON_I_R))	2	1–2	5-6-11
2	((NR,ISN_I_R NR,ISN_I_R) (NC,OSG_I_C NR,ISN_I_R))	2	1–6	6.5-5-3
3	(NC,KGU_I_C (NC,OSG_I_C NR,ISN_I_R))	2	1–2	8.5–3
4	(NC,STA_I_C (NC,OSG_I_C NR,ISN_I_R))	2	1–2	5–3.5
5	(NR,ISN_I_R (NC,STG_I_C NR,SON_I_R))	2	1–2	6.5–11
6	(NR,OSG_I_R (NR,ISN_I_R NR,ISN_I_R))	2	6–6	3–7
7	((NC,STA_I_C NC,OSG_I_C) NC,STG_I_C)	2	1–2	5–6
8	((NR,ISN_I_R NR,ISN_I_R) NC,OSG_I_C)	2	1–6	6.5–5
9	(NC NR,SON_I_R)	2	1–2	14.5
10	(NC,KGU_I_C NC,OSG_I_C)	2	1–2	8.5
11	(NC,OSG_I_C NR,ISN_I_R)	4	1-1-2-6	3
12	(NC,UMA_I_C NC,UMA_I_C)	2	2–3	1.5
13	(NR,DAB_I_R NC,STA_I_C)	2	5–5	4
14	(NR,ISN_I_R NC,STG_I_C)	2	1–2	6.5
15	(NR,ISN_I_R NR,ISN_I_R)	5	1-1-6-6-6	7
16	(NR,UMA_I_R NR)	2	1–3	5

*Note*. This table shows us with which techniques the -81 kg Olympic champion of the Rio Olympics scored. The identifier that differentiates each of the T-patterns detected is presented, its pattern in chain format—constitutive multi-events -, the number of occurrences that the T-pattern has and in which combats they take place, as well as the average of the internal intervals between the constituent multievents.

## Discussion

The JUTACTIC observation instrument has been designed, which provides evidence of validity, reliability and operability, following the criteria of the observational methodology [19], which allows characterizing the technical-tactical behaviour of *judoka* in combat. The observational system will be very useful for judo coaches by allowing them to observe, analyse and intervene in competition preparation from the study of their own *judoka*, their rival, or the interaction between *judoka*.

The observational instrument has been built at a technical level from a theoretical framework consolidated by the International Judo Federation in the SOR competition regulations [18]. The content validity of the observational instrument has been guaranteed by passing the precautionary test and based on expert judgment.

The reliability of the observational system developed has been guaranteed by the inter and intra-observer concordance results from the results obtained in the Fleiss Kappa coefficient [28] and Krippendorff Alpha.

Looking further at the data quality evidence, the generalizability design carried out, [Category] [Participants] / [Matches], is very relevant in this study; it allows us to answer the question: How many matches do we need from a *judoka* to capture with guarantees their technical-tactical performance in competition?

With regard to the operability of the observational system and the possibilities of practical application, firstly, the sample which configures each data packet represents the behaviour displayed by both *judoka* who face each other and their interaction; which supposes a relevant contribution of the present observation system in relation to other studies [5, 10, 12, 13, 17]. Each of the samples provides relevant diachronic information on what happened in the match, considering the multidimensionality of the observation instrument.

It must be said that the data obtained from the different observations directly provide the coaches with very useful information. Since seeing the document obtained from the LINCE PLUS program directly and without any type of statistical processing, it is possible to know the technical-tactical characteristics of the observed *judoka* and his rival. Note that the presented tool also provides time and movement information, it can be modified and adapted to other sports thanks to LINCE PLUS. Which shows great versatility.

Thus, it is very important for us to point out that this tool is very useful. Since the data they give us does say interesting information. In Table 4 (example of a record obtained by means of the observation instrument, corresponding to one particular match), the techniques used and the *kumi-kata* (grip) prior to said attacks can be seen in the different effort sequences. The situation on the *tatami* and the score obtained were also recorded. In said record we observe that at a technical level the rival performs *tai-otoshi*, *sode-tsuri-komi-ashi*, *de-ashi-harai* combined with *ko-uchi-gari* and *uchi-mata* during the match. All this in different sequences of effort throughout the fight. For his part, the champion performs *uchi-mata* twice, resulting in *ippon* and winning the match the second time he does it.

The robustness of the observation instrument allows us to use different techniques for analysing the records. Some studies have already combined different observational analysis techniques in combat sports [29]. Traditionally, descriptive analysis has focused a large part of the studies, which is why in our work we have made a brief description of the main results, on which their contributions can be explored in greater depth, to focus on a technique that offers great possibilities of detecting regular behaviour structures: T-patterns.

The informative potential of the T-patterns detected using the free software THEME (v.6 Edu) [22] is another good example of the possibilities of the observational instrument to influence relevant aspects in the opinion of the coach in the preparation for competition. Although judo combat incorporates throwing and grappling techniques, in this paper, the selected examples refer to throwing techniques. Specifically, and as an example, the results obtained when incorporating the dimensions: direction of attack in actions 1, 2, 3 or 4 in the detection of T-patterns, indicate that Khalmurzaev uses leg attack techniques against a right-handed opponent (*ashi-waza*), especially *sasae-tsuri-komi-ashi* on the right and *o-soto-gari* on the left. Furthermore, regular behaviour structures have been detected in which the Russian *judoka* is able to stop the attacks of his rivals with *ippon-seoi-nage*. Regularities have also been detected regarding attacks from both sides, both backwards and forwards, both from Khalmurzaev and from his rivals.

On the other hand, to study the technical actions used and their effectiveness, the dimensions have been activated: punctuation in the actions (from 1 to 4). All the T-patterns detected -whether from the champion or from the rivals- refer to actions that do not obtain a score -more frequent than those that do-. One option to favour the detection of these regular behavioural structures, in addition to expanding the observational sampling, is to group the different categories which imply scoring -*ippon*, *waza-ari* and *yuko*- of both the champion and the rival [30].

In the case of Khalmurzaev’s examples, we can see how the Russian *judoka* shows regular behaviour structures with different leg attacks (*ashi-waza*), especially *sasae-tsuri-komi-ashi* on the right side and *o-soto-gari* on the left side, with which a clear tendency can be seen to make his right-handed opponent react with attacks in different directions. In the case of the opponent a lot of *ippon-seoi-nage* attacks are made from the right, whatever action his opponent makes. In this case, he is left-handed and gripping up with his left hand is predominant. In relation to the effectiveness and with the data obtained in the form of patterns in chain format, it reveals that multiple attacks are made without scoring and, therefore, without effectiveness. This data, in our opinion, is relevant knowledge, although there is no score, what techniques are used by the *judoka* under study and how many times they attack with one or the other. Since although there is no effectiveness, it can be a strategy to make combinations or chains of other techniques. This is one of the reasons why we have studied the effort sequences as an interrelation between both *judokas*, recording all the attacks and combinations that were made in the same attack sequence.

With the information contained in the detected T-patterns (Tables 5 and 6) of the designed observation system, judo technicians and athletes will be able to obtain relevant information for the preparation of technical-tactical competitions, both for the athletes themselves and about the rivals they face, based on a systematic and rigorous analysis of the combat.

In the future, it would be interesting to carry out a study of the usefulness of the resource that we have presented in its application in sports training environments and to be able to consider whether its use can generate improvements in the performance of athletes.

## Conclusions

We consider that this work provides a valid and reliable tool for the observational analysis of judo, JUTACTIC, which can allow, through the subsequent analysis of the records made, one to know the technical-tactical profiles of the *judoka* analysed. It is not, therefore, only a tool applicable to research studies, but it could also be a good instrument for training control in its tactical and technical components. In fact, the present work does not exploit all the possibilities of analysis of the records made since we could apply multiple analysis techniques (T-Pattern, polar coordinates, lag sequential analysis…) in different orientations (analysis of the effectiveness, of the relationships between techniques…).
